# Comprehensive Analysis of Mycotoxins in Green Coffee Food Supplements: Method Development, Occurrence, and Health Risk Assessment

**DOI:** 10.3390/toxins17070316

**Published:** 2025-06-21

**Authors:** Laura Carbonell-Rozas, Octavian Augustin Mihalache, Renato Bruni, Chiara Dall’Asta

**Affiliations:** 1 Department of Food and Drug, University of Parma, 43124 Parma, Italy; augustinoctavian.mihalache@unipr.it (O.A.M.); chiara.dallasta@unipr.it (C.D.); 2 Dipartimento di Biologia Evolutiva e Funzionale, Sez. Biologia Vegetale e Orto Botanico, Università degli Studi di Parma, Parco Area delle Scienze 11A, 43100 Parma, Italy; renato.bruni@unipr.it

**Keywords:** mycotoxins, green coffee, food supplements, dietary supplements, risk assessment

## Abstract

This study investigates the presence of mycotoxins in green coffee-based dietary supplements to ensure their safety, given the potential risks of contamination and the growing interest in them among consumers. A sample treatment based on a salting-out assisted liquid–liquid extraction (SALLE) followed by one-step solid-phase extraction (SPE) was selected for the extraction and clean-up of 15 mycotoxins followed by ultra-high performance chromatography–tandem mass spectrometry detection (UHPLC-MS/MS). The target mycotoxins included aflatoxins (AFG1, AFG2, AFB1, AFB2), *Alternaria* toxins (AOH, AME, TEN), ochratoxin A (OTA), fumonisins (FB1, FB2), zearalenone (ZEN), trichothecenes (T-2, HT-2), enniatin B1 (ENNB1), and beauvericin (BEA). The proposed method was successfully characterized, obtaining high recoveries, a satisfactory precision, and low detection limits. Subsequently, the method was applied for the analysis of 16 commercial food supplements. The analysis revealed the presence of mycotoxins in all samples investigated with *Fusarium* mycotoxins as the most prevalent. The dietary exposure and risk characterization revealed a low level of risk, except for AFs where chronic exposure in adults may lead to potential health concerns.

## 1. Introduction

Food or dietary supplements are concentrated sources of nutrients or other substances with a nutritional or physiological effect such as mineral and vitamins, amino acids, fiber, and plant and herbal extracts (also known as “botanicals”) that are marketed in “dose” form (e.g., pills, tablets, capsules, liquids in measured doses) [1]. The global food or dietary supplements market is steadily growing in most countries. The USA, Europe, and Japan account for the largest share of the market, followed by Asia, Australia, and Oceania, all of which demonstrate the expansion of the market [2]. The World Health Organization (WHO) has estimated a growing demand for botanicals and their derived products at a rate of 15–25% per year, while the global dietary supplements market size accounted for USD 191.1 billion in 2020 and is expected to reach USD 307.8 billion by 2028 [3].

Green coffee, derived from unroasted coffee beans or green coffee beans (GCBs), is widely promoted for its health-promoting potential and is increasingly incorporated into dietary supplements due to its high content of bioactive compounds, including chlorogenic acids, caffeine, diterpenes, and soluble fiber [4]. Its properties are mainly related to antioxidant activity, but GCB extracts have also been marketed as weight loss supplements and to reduce glycemic response [5].

Nevertheless, GCBs, and consequently coffee products, are susceptible to mycotoxin contamination, with ochratoxin A (OTA) being one of the most prevalent and concerning toxins. [6]. OTA is primarily produced by *Aspergillus* and *Penicillium* species during post-harvest handling and storage under warm and humid conditions, and has frequently been detected in green coffee beans [7]. Due to its nephrotoxic, immunosuppressive, and potential carcinogenic effects, OTA contamination in coffee poses a significant food safety risk [8]. OTA has been classified as a possible carcinogen to humans (group 2B) by IARC and is regulated in many countries [9]. The European Union has revised the maximum permissible levels for OTA, setting limits at 3 µg kg^−1^ for roasted coffee beans and ground roasted coffee, and 5 µg kg^−1^ for soluble or instant coffee, as specified in Commission Regulation (EU) 2022/1370 [10]. Furthermore, several studies have reported the occurrence of other mycotoxins in coffee and related products, including aflatoxins, fumonisins, zearalenone, and trichothecenes, highlighting the broader scope of potential contamination beyond OTA [11,12]. The presence of multiclass mycotoxins in such foods poses a significant public health concern due to their wide-ranging adverse effects [13]. These mycotoxins can originate at various stages, from cultivation to storage, especially under conditions of high humidity and temperature [14,15]. In addition, commercial GCB-based dietary supplements often contain additional ingredients such as herbal and/or fruit extracts aimed at improving taste or enhancing bioactivities, increasing the possible co-occurrence of non-expected mycotoxins. Furthermore, the recourse to GCB extracts may determine an undesired concentration of mycotoxins in the final product even when the starting crude seeds are compliant with the legal limits.

The growing popularity of botanicals in Europe has led to the introduction of several regulations to safeguard public health and to ensure their quality, efficacy, and safety [16]. To ensure the safety and quality of GCBs and their related products, robust analytical methods are required for the simultaneous detection and quantification of multiple mycotoxins in such complex matrices. While most reported methods have focused on roasted coffee or coffee-based beverages [7,17,18], the analysis of green coffee has gained increasing attention in recent years due to its rising use in health products. However, the evaluation of mycotoxin contamination in GCB-based food supplements remains limited and underexplored considering their increased public interest [11,12], and there is currently no specific regulation governing the safety and quality of such products.

Techniques such as liquid chromatography–tandem mass spectrometry (LC-MS/MS) have emerged as the gold standard due to their high sensitivity, selectivity, and ability to analyze multiple residues in complex matrices. Additionally, sample preparation methods like QuEChERS (Quick, Easy, Cheap, Effective, Rugged, and Safe) and immunoaffinity column (IAC) clean-up are employed to minimize matrix interferences and enhance analytical accuracy [7]. However, these approaches can be time-consuming and costly, particularly in the case of IAC clean-up, due to the requirement for highly specific antibodies which limits the scope of the analysis. Despite advancements in analytical methods for GCBs and byproducts, challenges persist in the co-occurrence analysis of mycotoxins, the variability in contamination levels due to differences in coffee origin and processing methods, and the matrix complexity. Moreover, there is a growing need to address emerging mycotoxins and masked forms that may not be included in routine analysis methodologies [19].

The aims of this study were (i) to develop and validate a multi-mycotoxin analytical method based on a salting-out assisted liquid–liquid extraction (SALLE) combined with one-step solid-phase extraction (SPE) prior HPLC-MS/MS analysis; (ii) to investigate the occurrence of mycotoxins in commercial green coffee-based supplements available in the Italian Market; and (iii) to assess the potential health risk associated with their consumption by performing a preliminary dietary exposure assessment.

The targeted mycotoxins include both regulated compounds—such as aflatoxins (AFG1, AFG2, AFB1, AFB2), OTA, fumonisins (FB1, FB2), ZEN, and trichothecenes (T-2 and HT-2)—and currently unregulated, so-called “emerging mycotoxins,” such as *Alternaria* toxins (AOH, AME, TEN), enniatin B1 (ENNB1), and beauvericin (BEA). These mycotoxins were selected based on their known or suspected toxicity, documented occurrence in coffee or botanical supplements, and their inclusion in regulatory frameworks or as emerging contaminants of interest in food safety surveillance.

## 2. Results and Discussion

### 2.1. Clean-Up Selection

The QuEChERS technique has been applied for multimycotoxin analysis and is widely used in the investigation of coffee and related products, as summarized in Table S1. Normally it involves a first step based on a salting-out assisted liquid–liquid extraction (SALLE). SALLE offers a straightforward and time-efficient approach for extracting target analytes from complex matrices. The salting-out phenomenon promotes the partitioning of analytes into the organic phase by reducing the miscibility between water and polar organic solvents through salt addition. This results in phase separation under suitable conditions. The method is further distinguished by its minimal solvent requirements, operational simplicity, and compatibility with conventional laboratory instrumentation. The hydroalcholic mixture composed of acetonitrile (MeCN) and water (50:50, *v*/*v*) appears to be the most widely adopted extraction solvent, providing consistently good recoveries. Extraction solvents are often acidified with 1% formic or acetic acid to enhance the extraction of analytes that, at neutral pH, would exist in a dissociated form and thus may not be efficiently extracted by non-polar solvents.

SALLE is often combined with clean-up techniques such as dispersive solid-phase extraction (dSPE) or solid-phase extraction (SPE), particularly when analyzing complex matrices. In the case of coffee samples, dSPE has been widely reported as a clean-up step, mainly using MgSO_4_ and C18 as the salting-out agent and dispersive sorbent, respectively. Nevertheless, the potential loss of analytes, which can be retained in the sorbent, limits the use of other sorbents that are more selective. On the other hand, although higher reproducibility and selectivity are attributed to SPE, it requires a longer preparation time due to the pre-conditioning of the employed cartridges. In addition when SPE is based on IAC clean-up, the high selectivity of the selected column, mainly for OTA, limits the analysis to a few compounds [20,21].

In this study, we proposed the comparative evaluation of two different clean-up procedures based on dSPE and SPE, after a traditional SALLE employing an aqueous solution containing 1% formic acid and MeCN as the extraction sovlent which was scaled-down in order to reduce organic solvent consumption. Thus, 5 mL of the acidic aqueous solution and 5 mL of MeCN were used, instead of the 10 mL that is commonly used for both solvents in this approach [22]. Consequently, the addition of salts was also scaled-down. Thus, 2 g of MgSO_4_ and 0.5 g of NaCl were added to favor phase separation. After mechanical agitation and centrifugation, the 4 mL of the supernatant was collected and purified via two clean-up methods including dSPE and SPE techniques. (1) As for the dSPE, the organic solution was transferred to a 15-falcon tube containing 180 mg of MgSO_4_ and 60 mg of C18. The corresponding amounts were selected, scaling-down the amount used in a previous work considering the reduction in the sample size in this study (1 g) [22]. The mixture was agitated in a mechanical shaker for 10 min, and centrifuged for 10 min. The resulting supernatant was collected and dried under a gentle nitrogen stream. The dry extract was redissolved in 1 mL of MeOH/water (75:25, *v*:*v*), and filtered with a glass fiber filter (1.2 µm pore size, 13 mm diameter) prior its analysis by LC-MS/MS.

(2) As for SPE, a Waters^®^ HLB PRIME SPE cartridge, which does not require conditioning prior to the charge of the sample, was considered. In order to develop a simple and rapid protocol, the cartridge was employed in a single-step SPE process, in which the sample is passed directly through the cartridge, allowing for the retention of interferences and impurities while permitting the elution of the target compounds. This approach also allowed us to reduce the volume of toxic solvents, usually involved in a traditional SPE. Thus, the 4 mL supernatant was passed through the SPE cartridge and the eluted portion was collected in a glass vial for its subsequent dryness under a nitrogen stream. As for (1), the dry extract was redissolved in 1 mL of MeOH/water (75:25, *v*:*v*), and filtered with a glass fiber filter (1.2 µm pore size, 13 mm diameter) prior its analysis by LC-MS/MS.

**Figure 1:**
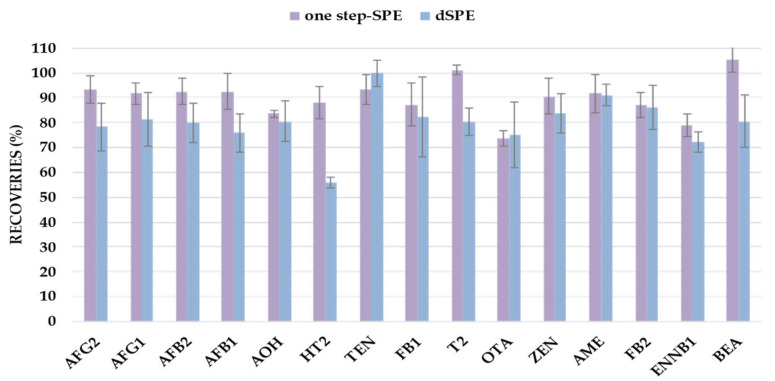
Clean-up approaches’ comparison in terms of recoveries. Error bars represent relative standard errors (n = 4).

In both cases, to evaluate the procedures in terms of recovery, a blank representative sample of a green coffee dietary supplement was fortified prior to sample treatment and compared with a corresponding sample that was subjected to each procedure and spiked at the end. Samples were processed and injected in duplicate (n = 4). Figure 1 compares the recovery values for each mycotoxin obtained using the SALLE followed by dSPE or one-step SPE as clean-up procedures.

As observed, the one-step SPE procedure consistently yielded higher recoveries across all target mycotoxins, with a particularly notable improvement in the recovery of H-T2, which was below 60% when using dSPE as the clean-up step. In contrast, the SPE approach achieved recoveries above 80% for all analytes. The significant enhancement in HT-2 recovery was a key factor in selecting the one-step SPE protocol. Additionally, matrix effects were reduced when using SPE, further supporting its suitability for complex coffee-based matrices, likely due to a more effective removal of medium-polar interfering compounds that are naturally present in the green coffee matrix.

These results underscore the superior efficiency of the proposed method compared to previously reported sample preparation strategies for the extraction and purification of multiclass mycotoxins in coffee-based matrices (Table S1). Therefore, based on both the analytical performance and the simplicity of implementation, the one-step SPE clean-up was selected for subsequent analyses.

**Figure 2:**
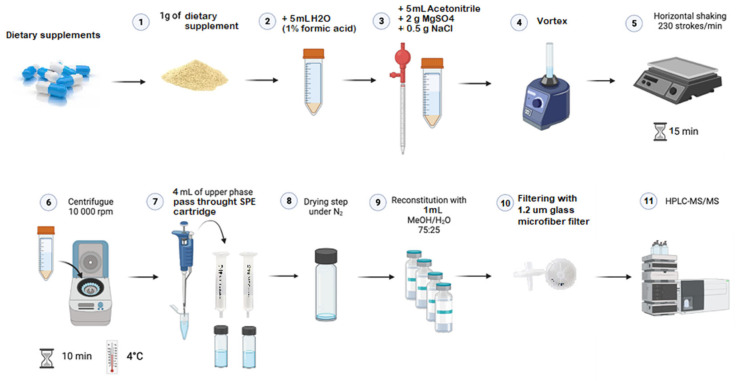
Scheme of the proposed sample treatment procedure.

The schematic representation of the sample treatment procedure—based on SALLE followed by one-step SPE clean-up—as a key step within the analytical workflow is illustrated in Figure 2.

When compared to previously reported methods, it is worth noting that although SPE has been conducted as a clean-up stage for similar samples, using other types of cartridges such as Bond Elut or Waters^®^ Oasis MAX [11,12], these typically require pre-conditioning, washing, and elution steps. In contrast, the proposed method using the Waters^®^ HLB PRIME SPE (Waters Corporation, Milford, CT, USA) cartridge eliminates these stages, thereby reducing both operational time and solvent consumption. Furthermore, other methodologies based on the modified QuECheRS technique proposed the use of an additional step using hexane [11,22] involving a high volume of toxic organic solvents. In this regard, the proposed method represents a more sustainable alternative to previously reported methodologies for the extraction of multiclass mycotoxins from green coffee and related products. It aligns with emerging trends in Green Sample Preparation (GSP) principles [23], aiming to minimize the environmental impact of analytical procedures and reduce operator exposure to hazardous substances.

### 2.2. HPLC-MS/MS Method Optimization

The HPLC-MS/MS method employed in this study was adapted and further improved from a previously optimized protocol for the multiresidual quantification of mycotoxins [24]. Several mycotoxins, including T-2, HT-2, ENNB1, ZEA and BEA, were incorporated into the method to expand the analytical scope and enhance the comprehensiveness of the study in the selected samples. Some of these compounds are considered ‘emerging mycotoxins’, whose presence in novel food products such as food supplements is of growing concern due to their potential health risks and lack of regulatory limits.

To optimize MS parameters for the target mycotoxins, individual standard solutions (100 µg/mL) were infused into the ion source at 5 µL/min simultaneously with an isocratic mobile phase (MeOH:H_2_O, *v*/*v*) at a flow rate of 0.3 mL/min. The most intense precursor ion and two corresponding product ions were carefully selected and optimized for each analyte under selective reaction monitoring (SRM) conditions, along with their optimal collision energies. The electrospray ionization (ESI) source was operated in both positive and negative modes to determine the ionization conditions that produced the most intense signal for each compound. Source voltages, gas flows, and temperatures were selected based on the least sensitive compounds in order to favor their ionization. The SRM transitions and their corresponding collision energies are shown in Table 1**.** Protonated [M + H]^+^ and deprotonated [M − H]^−^ adducts were monitored as precursor ions depending on the analyte. In all cases, two ion transitions were measured, the most abundant for quantification (Q) purposes and the second one for peak identification (I) and confirmation.

Two chromatographic columns, a C18 Kinetex column (100 mm × 2.1 mm, 2.6 μm) and a SunShell C18 Column (100 mm × 2.1 mm, 2.6 µm), were tested using a mobile phase consisting of ultrapure water with 0.2% acetic acid and 5 mM ammonium acetate aqueous solution (solvent A) and MeOH with 0.2% acetic acid as the organic solvent (solvent B). Although similar separation was achieved, the SunShell C18 column provided better peak shapes, so it was selected for further experiments. The gradient was optimized until peak-to-peak resolution was achieved for all mycotoxins. Thus, the separation of the 15 target mycotoxins was achieved in less than 12 min.

### 2.3. Method Characterization

The proposed SALLE–SPE–HPLC–MS/MS method was validated based on key performance parameters, including linearity, limits of detection (LODs), limits of quantification (LOQs), and precision—evaluated as both repeatability and intermediate precision—as well as recovery rates (%R). In all cases, green coffee beans were used as a representative blank matrix due to the difficulty in sourcing a food supplement product that is free of the target mycotoxins.

Matrix-matched calibration curves were established by applying the full sample treatment procedure to blank samples in duplicate and subsequently fortifying the final extracts with the target mycotoxins at different concentration levels (1, 5, 10, 25, 50, and 100 µg/kg) which were also injected in duplicate. The peak area was considered as a function of the analyte concentration. LODs and LOQs were set as the lowest level of the calibration range, provided that these presented a S/N that is equal to or higher than 3 and 10, respectively (Table S3). Good linearity was observed with the coefficient of determination (R2) above 0.98 in all cases. Most of the target mycotoxins presented a LOQ of 1 µg/kg (except for AME and T2 with LOQ of 5 µg/kg). Good linearity was observed with the coefficient of determination (R2) above 0.98 in all cases. Most of the target mycotoxins exhibited LOQs of 1 µg/kg, with the exception of AME and T2 that showed slightly higher LOQs of 5 µg/kg. Given the absence of specific regulations for GCB or green coffee-based food supplements, the LOQs were compared with the established maximum limits for these compounds in relevant food commodities [25]. Notably, the LOQ for OTA was below the regulatory limit of 3 µg/kg set for roasted coffee beans and ground roasted coffee products.

Matrix effects (MEs) were evaluated by comparing the slopes of solvent-based and matrix-matched calibration curves. Signal suppression was observed in all cases; however, the use of matrix-matched calibration effectively compensated for these effects.

Trueness was assessed by recovery experiments at two concentration levels within the method linear range (5 and 50 µg/kg). It was calculated as the ratio between the peak area of samples spiked before the sample treatment and the peak area of the samples fortified after sample treatment at the corresponding concentration. Samples were processed in triplicate in each study level. The average recovery is shown in Table S4, and in all cases, it was between 70 and 110%. These results demonstrated that the proposed SALLE–one-step SPE could be satisfactorily applied for the extraction and isolation of the target mycotoxins of GCBs and byproducts.

The precision of the method was measured in terms of repeatability (i.e., intra-day precision) and intermediate precision (i.e., inter-day precision). Precision was assessed by the relative standard deviation (RSD, %) for each spiking level (5 and 50 µg/kg) (Table S4). In both cases, RSD was under 20%, fulfilling the recommendation of the guidance for the analysis of mycotoxins in foodstuffs [26].

### 2.4. Occurrence Study of Commercial Samples

**Figure 3:**
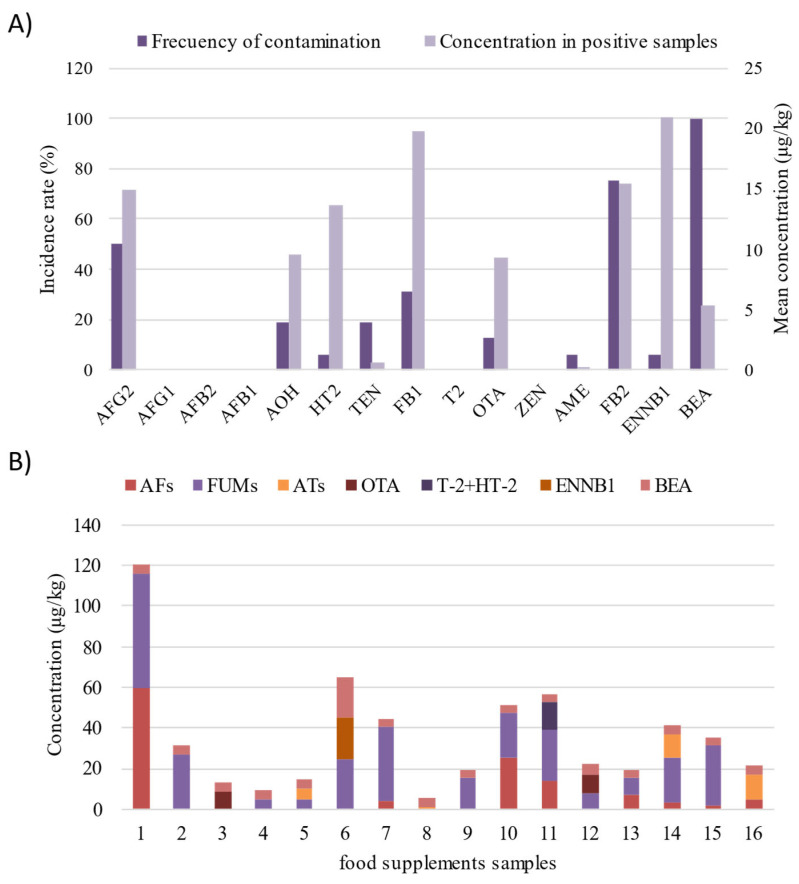
Overview of the co-occurrence and concentration levels of the target mycotoxins identified in green coffee-based food supplements. (**A**) Incidence rate (%, left axis) and toxin average concentration (μg/kg, right axis). (**B**) The total mycotoxin content, highlighting the specific mycotoxin class detected in each sample.

For the identification of mycotoxins in the commercial food supplements, the recommendations of the SANTE guideline were followed [27]. Samples were considered positive for a given mycotoxin when they met the following criteria: (i) a signal-to-noise (S/N) ratio of at least 3 for the target peak; (ii) relative ion intensities of the quantifier and qualifier ions that are consistent with those observed in standard solutions; and (iii) a calculated concentration exceeding the corresponding LOQ. The results are presented in Table S5. When mycotoxins were detected but not quantified because their concentrations were below the LOQs, they were reported as <LOQ. In cases where the mycotoxins were not detected, the <LOD was used. Figure 3 provides an overview of the co-occurrence and concentration levels of the target mycotoxins identified in the analyzed samples.

Figure 3A shows the incidence rates (left axis) and the average concentration of each mycotoxin found to be positive (right axis) across all green coffee-based food supplements under study. BEA was the most frequently detected, appearing in 100% of the samples followed by FB2 and AFG2, which were present in 75% and 50% of the samples, respectively. Although BEA was the most frequently detected mycotoxin, its concentration remained relatively low, averaging approximately 5 µg/kg. In contrast, although the incidence for ENNB1 was really low since it was only present in one of the investigated samples, the found concentration reached 20 μg/kg. This concentration was followed by FUMs for which an average concentration of 19 and 15 μg/kg was observed for FB1 and FB2, respectively. Although AFG2 reached an average concentration of 14 due to the presence of a maximum value of 59 μg/kg in one of the samples, the occurrence of other AFs was not confirmed. Similarly, other mycotoxins such as T2 or ZEN were not detected. However, HT-2 toxin, a major metabolite of T-2, was detected in approximately 6% of the samples, with an average concentration of 13.7 μg/kg. This finding may be attributed to the rapid metabolism of T-2 toxin into several metabolites, among which HT-2 is considered the primary derivative. Overall, it was observed that Fusarium-produced mycotoxins, such as FUMs, ENNB1 and BEA, were the most predominant mycotoxins found in the analyzed samples. Given the increasing occurrence of ENNB1 and BEA in plant-based food supplements and their emerging toxicological significance, their presence raises concerns regarding potential cumulative exposure and underscores the need for further toxicological and regulatory evaluation [28]. To date, no definitive conclusions have been established regarding the health risks associated with chronic exposure to ENNs or BEA, primarily due to insufficient in vivo toxicity data [29].

Although OTA is frequently reported in coffee products, it was detected in only 12.5% of the samples in this study. However, its average concentration of 9.3 μg/kg exceeded the maximum limits established for comparable food matrices. This elevated level may be attributed to the absence of a roasting process in GCBs, as roasting is known to significantly reduce OTA content.

As shown in Figure 3B, which illustrates the total mycotoxin content per sample and highlights the specific classes of mycotoxins detected, all food supplement samples were found to be contaminated with at least one mycotoxin. The variability observed among samples may be attributed to differences in the composition of the food supplements, including variations in the type, quality, and proportion of GCB extract used. Notably, Sample 1 exhibited particularly high contamination levels, with significant concentrations of both. In general, it is worth mentioning that most of the samples presented mycotoxins from different families, highlighting the widespread co-occurrence of multiple mycotoxins and the need to monitor them simultaneously.

### 2.5. Dietary Exposure and Risk Characteriation

**Figure 4:**
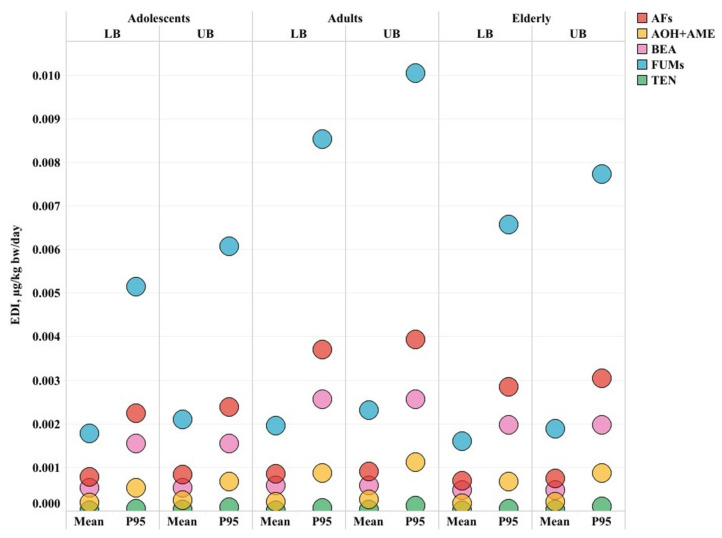
Estimated daily intake (EDI) of mycotoxins based on the consumption of green coffee-based supplements; LB = lower bound; UB = upper bound; Mean and P95 = mean and high consumption.

**Figure 5:**
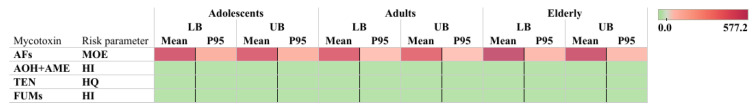
Risk characterization considering the individual and cumulative exposure to mycotoxins based on the consumption of green coffee-based supplements; LB = lower bound; UB = upper bound; Mean and P95 = mean and high consumption; HQ = hazard quotient; HI = hazard index; MOE = margin of exposure; red = potential health concern; green = tolerable exposure levels.

The estimated daily intakes (EDIs) of each mycotoxin per consumer group considering the consumption of green coffee-based supplements in a lower and upper bound scenario with mean and high consumptions are presented in Figure 4 and Figure 5.

The EDI of AFs based on the consumption of green coffee-based supplements was very low, between 0.0007 and 0.004 μg/kg bw/day. Comparable results have been previously found where the exposure to AFs from consumption of green coffee was at 0.00003 μg/kg bw/day [12] while plant-based supplements led to an EDI of 0.11 μg/kg bw/day, indicating an exposure that is 27 times higher than ours [30].

Despite the low exposure levels, the MOEs were between 101 and 577 (<10,000), indicating a potential health concern even in the lower bound (optimistic) scenario for all three consumer groups. The International Agency for Research on Cancer (IARC) has classified aflatoxin B1 (AFB1) as a Group I carcinogen, indicating that it is carcinogenic to humans, and ranks the toxicity of aflatoxins in the following order: AFB1 > AFG1 > AFB2 > AFG2 [9]. In light of our findings, even low levels of dietary exposure to aflatoxins may pose a potential health risk.

For *Alternaria* toxins, the highest exposure was for adults at 0.001 μg/kg bw/day, indicating low exposure levels even for high consumers. Our results indicate a higher exposure than what was previously reported for the consumption of green coffee (0.00006 μg/kg bw/day) [12]. BEA exposure levels were between 0.0004 and 0.002 μg/kg bw/day. Similar exposure levels were also reported in Switzerland (0.0013 μg/kg bw/day) [12]. The highest EDIs were observed for FUMs from high consumers of supplements ranging from 0.001 to 0.010 μg/kg bw/day. Our EDIs are lower than those previously reported by other studies with exposure levels at 0.02 μg/kg bw/day [12].

The low exposure levels for *Alternaria* toxins and FUMs are well below the health-based guidance values (highest HI = 0.4 for AOH + AME), indicating a negligible level of risk even for the high consumption of green coffee-based supplements (Figure 5).

Overall, the highest exposure to mycotoxins is for adults followed up by adolescents, and the elderly. Exposure to mycotoxins indicates a low level of risk, except for AFs where chronic exposure may lead to potential health concerns.

## 3. Conclusions

This work presents a reliable and efficient analytical method for the determination of multiclass mycotoxins in green coffee-based food supplements, combining SALLE with a one-step SPE clean-up, followed by UHPLC-MS/MS. The reduction of the organic solvent consumption during the extraction along with the simplification of the traditional SPE makes the proposed method a more sustainable and practical alternative to the previously reported methodologies for similar purposes.

The analysis of commercial green coffee-based supplements revealed the presence of at least one mycotoxin in all tested samples, with Fusarium-produced mycotoxins as the most prevalent. It is worthy to mention that BEA, which is considered as an emerging mycotoxin, was present in all investigated samples. Although OTA and AFs were less frequently detected, their concentration in some cases exceeded regulatory limits for similar food matrices, underscoring the potential risk associated with their consumption. In this framework, the preliminary dietary exposure assessment confirmed a generally low risk for most detected mycotoxins. However, the estimated MOEs for AFs, particularly for AFB1, were below the safety threshold even at low exposure levels, suggesting a potential health concern with chronic consumption. This is particularly relevant given the lack of specific regulatory limits for green coffee-based supplements. Nevertheless, following this initial approach, the investigation of these products can be expanded with a larger number of samples, enabling a better understanding of their contamination levels and related risks, including the occurrence of unexpected mycotoxins.

Overall, these findings underscore the need for controlling these under-investigated products and the establishment of regulatory oversight in the dietary supplement industry, especially for botanical-based products such as green coffee. In this context, the proposed analytical method offers a practical tool for the routine monitoring and safety assessment of green coffee food supplements.

## 4. Materials and Methods

### 4.1. Reagents and Materials

Analytical standards of aflatoxin B1, B2, G1, and G2 were purchased from Fermentek Ltd. (Jerusalem, Israel), while Alternaria mycotoxins (AOH, AME, TEN) were obtained from Biopure (Getzersdorf, Austria). FUM standard solutions as well as ZEN, OTA, T-2 HT-2, ENNB1, and BEA were purchased from Romer Laboratories (Tulln, Austria).

Ultra-pure water and methanol (MeOH) were purchased from VWR International (Milano, Italy), acetonitrile (MeCN) from Sharlab (Barcelona, Spain). Acetic acid, ammonium acetate, sodium chloride, and magnesium sulfate anhydrous were supplied by Sigma-Aldrich (Milan, Italy).

### 4.2. Sample Collection

Any green coffee dietary supplement produced in different European countries and available for sale in Italy during October 2023 was considered eligible. A total of 16 green coffee-based dietary supplement samples—available in tablet, capsule, and, in one case, liquid form—that were marketed in Italy were purchased from the online market. To ensure sample homogeneity, the tablets were ground and capsules were decapsulated, with multiple portions taken from each unit to obtain a representative sample. Then, the samples were stored at a temperature of 4 °C and kept at room temperature until processing.

Details on the supplement formulations, including active ingredients, excipients, dosage, unit weight, and label information, are provided in Table S2.

### 4.3. Sample Treatment: SALLE + One Step-SPE

A sample of 1 g of the homogenized powder of the dietary supplement was mixed with 5 mL of an aqueous solution containing 1% formic acid, followed by 5 mL of MeCN. Subsequently, salts were added—2 g of MgSO_4_ and 0.5 g of NaCl— to favor salting-out, and the mixture was immediately vortexed for 1 min. Then, the samples were placed in a horizontal shaker (Ika-werke, Staufen im Breisgau, Germany) at 230 oscillations per minute for 15 min. The samples were then centrifuged at 10,000 rpm for 10 min at 4 °C (5810 R centrifuge, Eppendorf, Hamburg, Germany). A total of 4 mL of the upper layer was collected and passed through a Waters^®^ HLB PRIME SPE cartridge, which requires neither conditioning nor additional solvents for mycotoxin elution. The 4 mL eluate was collected in a glass vial and evaporated to dryness under a gentle stream of nitrogen (N_2_). The residue was first reconstituted with 1 mL of methanol/water (75:25, *v*/*v*), then filtered using a 1.2 µm glass microfiber filter and transferred to a 1.5 mL vial for its injection in the UHPLC-MS/MS system.

### 4.4. LC-MS/MS Conditions

The analysis was performed on a UHPLC Dionex Ultimate 3000 separation system coupled to a triple quadrupole mass spectrometer (TSQ Vantage; Thermo Fisher Scientific Inc., San Jose, CA, USA) equipped with an electrospray source (ESI).

Briefly, the chromatographic separation was performed on a SunShell C18 Column (100 mm × 2.1 mm, 2.6 µm) using a mobile phase consisting of ultrapure water (VWR International, Milan, Italy) with 0.2% acetic acid (Sigma-Aldrich, Milan, Italy) and 5 mM of ammonium acetate (Sigma-Aldrich, Milan, Italy) as aqueous solvent A and MeOH with 0.2% acetic acid as organic solvent B. The eluent gradient profile was as follows: 0–1 min 5% B; 1–8 min 5–90% B; 8–11 min 90% B; 11–13 min 90–5% B and 13–18 min 5%, using 0.4 mL/min as flow rate. The column temperature was set at 40 °C and the injection volume was 3 µL.

The mass spectrometer operated in positive electrospray ionization (ESI+) mode for the determination of AFB1, AFB2, AFG1, AFG2, FB1, FB2, TEN, and OTA, while negative electrospray ionization (ESI-) was used for AOH, AME, and ZEN. All the compounds were analyzed under SRM conditions which are shown in Table 1. Regarding the MS parameters for the analysis, the spray voltage was set at 3500 V, while the temperature of the capillary and of the vaporizer were set at 270 °C and 200 °C, respectively. The sheath gas flow was set at 50 units, and the auxiliary gas flow was set at 5 units.

Chromeleon HPLC and Xcalibur (version 4.1) software (Thermo Fisher Scientific) were used for acquisition and data analysis, respectively.

### 4.5. Food Consumption Scenarios

Botanical supplements’ consumption data was retrieved from the latest Italian Food Consumption Survey (IV SCAI CHILD 2017–2020). We assumed these consumption data for our exposure modeling using occurrence data for green coffee-based supplements. We have taken into account the consumption for adolescents (10–17 years old), adults (18–64 years old), and the elderly (65–74 years old). The mean and high (95th percentile) consumption data are presented in Table S6.

### 4.6. Exposure Assessment and Risk Characterization

Before assessing Italian consumers’ dietary exposure, we treated the left-censored data (data below LOD and/or LOQ) using the substitution method as indicated by the European Food Safety Authority (EFSA) [31]. Therefore, we created two exposure scenarios, lower bound (LB) and upper bound (UB) scenarios, indicating a conservative and a less conservative exposure estimate. In the LB, the data below LOD and/or LOQ are replaced by 0 while in the UB, the data below LOD and/or LOQ are replaced with the instrumental values of the LOD and LOQ.

The estimated daily intake (EDI) of each mycotoxin was calculated using Equation (1):

EDI (μg/kg bw/day) = (C × FC)/bw)/1000(1)

where C = the average concentration of the mycotoxins found in the food items (µg/kg), FC = food consumption in g/day, and bw = bodyweight (55 kg (adolescents), 70 kg (adults and the elderly)) [32].

To assess if the exposure to mycotoxins indicates a potential health risk, we compared the EDI with health-based guidance values (HBGVs). For genotoxic and carcinogenic mycotoxins such as Afs, we used the margin of exposure (MOE) approach. The EFSA set a tolerable daily intake (TDI) of 1 μg/kg bw/day for FUMs due to an increased incidence of megalocytic hepatocytes in mice [33]. For the *Alternaria* mycotoxins, the EFSA recommended the use of a threshold of toxicological concern (TTC) due to the lack of toxicological data. The TTC for genotoxic *Alternaria* mycotoxins (AOH, AME) is 0.0025 µg/kg bw/day, while for the non-genotoxic *Alternaria* mycotoxin (TEN), the TTC = 1.5 µg/kg bw/day [34]. For Afs, we used the MOE approach to check for any potential health concerns. The MOE represents the ratio between the benchmark dose lower limit (BMDL) of the chemical (AFs) and the EDI of the consumers as shown in Equation (2):

MOE = BMDL10/EDI(2)

The BMDL10 for AFs is 0.4 µg/kg bw/day based on the incidence of hepatocellular carcinoma in rats [35]. When the MOE is lower than 10,000, it indicates a low health concern, while an MOE equal to or higher than 10,000 indicates a potential health concern from a public health point of view [36].

For the individual and cumulative risk characterization of FUMs and *Alternaria* mycotoxins, we used the hazard quotient (HQ) and the hazard index (HI), respectfully. The HQ is equal to the ratio between the EDI and the HBGV of the mycotoxin (Equation (3)):

HQ = EDI/HBGV(3)

The HI is the sum of the HQs of each mycotoxin (i.e., FB1 + FB2) used for a cumulative assessment (Equation (4)). If the HQ or HI are higher than 1, it indicates a non-tolerable level of exposure with potential health risks for the consumers [37].

HI = Σ HQs(4)

## Figures and Tables

**Table 1:** MS/MS conditions for the determination of the multiclass target mycotoxins.

Mycotoxins	RT (min)	Molecular Ion	Precursor Ion (*m*/*z*)	Product Ions (*m*/*z*)	Collision Energy (eV)
**AFG2**	6.94	[M + H]^+^	331.3	285 (Q)	30
				313 (I)	30
**AFG1**	7.16	[M + H]^+^	329	243 (Q)	25
				311 (I)	20
**AFB2**	7.36	[M + H]^+^	315.2	259 (Q)	30
				287 (I)	25
**AFB1**	7.58	[M + H]^+^	313.1	285 (Q)	25
				241 (I)	42
**AOH**	8.31	[M − H]^−^	257	213 (Q)	25
				215 (I)	29
**HT-2**	8.5	[M + H]^+^	442.3	263.1 (Q)	12
				164.1 (I)	26
**TEN**	8.65	[M + H]^+^	415	312 (Q)	20
				302 (I)	13
**FB1**	8.70	[M + H]^+^	722.4	334 (Q)	43
				352 (I)	42
**T-2**	8.98	[M + H]^+^	484.38	215 (Q)	21
				185 (I)	22
**OTA**	9.01	[M + H]^+^	404.5	238.7 (Q)	21
				101.7 (I)	68
**ZEN**	9.22	[M − H]^−^	317	131 (Q)	32
				175 (I)	32
**AME**	9.48	[M − H]^−^	271	256 (Q)	25
				213 (I)	41
**FB2**	9.55	[M + H]^+^	706.4	318 (Q)	42
				354 (I)	42
**ENNB1**	10.82	[M + H]^+^	640.6	196.2 (Q)	29
				186.2 (I)	37
**BEA**	11.23	[M + H]^+^	801	262 (Q)	54
				244 (I)	36

## Data Availability

The original contributions presented in this study are included in the article/Supplementary Materials. Further inquiries can be directed to the corresponding author.

## Supplementary Materials

The following supporting information can be downloaded at: https://www.mdpi.com/article/10.3390/toxins17070316/s1, Table S1: Overview of methods reported in the literature for the determination of mycotoxins in coffee and related products, including their main characteristics.; Table S2. Main characteristics of the green coffee-based nutraceuticals used in this study; Table S3. Statistical and performance characteristics of the proposed method to determine mycotoxins in green coffee-based nutraceutical samples; Table S4. Recovery and precision assays of the proposed method at two levels of concentration (5 and 50 μg/kg).; Table S5. Occurrence study of mycotoxins in green coffee-based food supplements. Results of positive samples (concentration of mycotoxin higher than LOQ. RSD (%), n = 3); <LOQ (detected but not quantified) and <LOD (non detected). Table S6. Consumer groups and mean and 95th percentile (P95) daily herbal based-supplements/nutraceuticals consumption in Italy. The supplementary materials refer to the references [11,12,20,21,22,38,39,40,41,42,43].
