# Supplementary material for: Comprehensive Analysis of Mycotoxins in Green Coffee Food Supplements: Method Development, Occurrence, and Health Risk Assessment
Source: Toxins (Basel). 2025 Jun 21;17(7):316. doi: 10.3390/toxins17070316 (PMC12298769; doi:10.3390/toxins17070316)
Supplement: Supplementary file 1 [file toxins-17-00316-s001.zip › toxins-3700422-supplementary.pdf]

toxins

MDPI

Supplementary Material: Comprehensive Analysis of Mycotoxins in Green Coffee Food Supplements: Method Development, Occurrence, and Health Risk Assessment

Laura Carbonell-Rozas, Octavian Augustin Mihalache, Renato Bruni, Chiara Dall'Asta

Table S1. Overview of methods reported in the literature for the determination of mycotoxins in coffee and related products, including their main characteristics.

Analyte	Matrix	Sample treatment	Separation & detection	% R	LOQ (µg kg^{-1})	Ref
OTA	Roasted coffee	SLE with MeOH:$NaHCO_3$ (1:1, *v*/*v*) and SPE with IAC (Ochraprep®)	HPLC-FLD	95-109	0.27-0.50	[1]
OTA	Instant coffee	SLE with hot water and SPE with IAC (OchraTest)	UHPLC-QqQ-MS/MS	85-117	1.73 pg mL^{-1}	[2]
CIT, OTA	Green coffee and dietary supplements	OTA: MeCN:water (60:40, *v*/*v*) Ochraprep® CIT: SLE + CitriTest	HPLC-FLD	>83.6 >74	0.36 2	[3]
OTA 2′R-OTA	Bean, roasted and instant coffee	SLE with MeOH/$NaHCO_3$ and SPE with IAC (OchraTest)	HPLC-FLD	81–89	0.3	[4]
AFB1, AFB2, AFG1, AFG2, OTA	Cocoa, coffee	Traditional QuEChERS and SPE with IAC (AFLAOCHRA prep)	HPLC-MS/MS	88-104	0.5-2.5	[5]
AFs, ENNs, ATs, FUMs, OTA, STC, ZEN, BEA	Coffee	Modified QuEChERS with MeCN and dSPE with C_{18} + SLE with hexane as second clean up	HPLC-MS/MS	> 65	0.1-50	[6]
AFs and OTA	Raw coffee beans	Modified QuEChERS method with little sample preparation excluding clean-up	UHPLC-MS/MS	63-89	0.45 to 1	[7]
ALT, AOH, AME, TEN, TeA	Green coffee	Modified QuEChERS with Na_2SO_4/NaCl (4 g + 1 g) and hexane	UHPLC-MS/MS	75-119	0.5-2.5	[8]
OTA, FB2, FB4, FB6	Roasted, green, instant coffee	Modified QuEChERS with acidic MeCN and SPE as clean up (Oasis MAX)	UHPLC-QqQ-MS/MS	>75 except for FB2 (40)	0.46-2 3.2-13	[9]
31 mycotoxins	Green coffee	Modified QuEChERS with acidic MeCN and clean up by: 1)SLE with hexane	HPLC-QqQ-MS/MS	82-117	0.5-10	[10]

		2)SPE cartridge (Bond Elut)				
34 mycotoxins	Green coffee-based dietary supplements	Modified QuEChERS with acidic MeCN	UHPLC-MS (QqQ and IT)	75-109 except for FUMs (50)	2.5-100	[11]

Table S2. Main characteristics of the green coffee-based nutraceuticals used in this study.

Sample Code	Type	Declared Content	Recommended Dose	Single Unit Weight
**1**	Capsules	Per 3 capsules: green coffee extracts 1500 mg, chlorogenic acid 675 mg, caffeine 45 mg	3 capsules per day after meals with plenty of water	1.9445 g
**2**	Capsules	Per 2 capsules: green coffee seed dry extract 500 mg	2 capsules per day after lunch	0.7356 g
**3**	Liquid	Per 20 mL: inulin 500 mg, green coffee extract 300 mg, chlorogenic acid 150 mg, orthosiphon extract 50 mg, pilosella extract 30 mg, pineapple extract 30 mg, dandelion extract 20 mg, artichoke extract 20 mg	20 mL per day diluted in 500 mL of water or other liquid to be taken in the morning	
**4**	Capsules	Per 1 capsule: green coffee extract 200 mg (of which chlorogenic acid 92 mg, caffeine 4 mg), choline bitartrate 104 mg (of which choline 41.65 mg), green tea extract 100 mg (of which epigallocatechins 16 mg, caffeine 10 mg), chromium picolinate 26 µg (of which iodine 11.5 µg), caffeine 29.5 mg	2 capsules per day	1.2520 g
**5**	Capsules	Per 3 capsules: green coffee extract 420 mg	3 capsules per day with a glass of water	1.8554 g
**6**	Tablets	Per 3 tablets: green coffee extract 600 mg (of which chlorogenic acid 270 mg), cassia nomame extract 255 mg (of which catechins 20 mg)	3 tablets per day before meals with a glass of water	1.2473 g
**7**	Tablets	Per 1 tablet: green coffee extract 400 mg (of which chlorogenic acid 200 mg)	1 tablet per day with plenty of water	0.5027 g

**8**	Filter Sachets	Per 2 sachets: green coffee seeds 1200 mg, green tea leaves 1200 mg, peppermint leaves 450 mg	1–2 cups (approx. 150 mL) of herbal tea per day, away from meals	1.7891 g
**9**	Capsules	Per 2 capsules: green coffee extract 606 mg, chlorogenic acid 273 mg	1 capsule before lunch and 1 capsule before dinner with plenty of water	0.9213 g
**10**	Capsules	Not Available	Not Available	0.6613 g
**11**	Capsules	Per 1 capsule: Maté extract 150 mg, green coffee extract 100 mg, total caffeine 9.5 mg	1 capsule in the morning on an empty stomach with plenty of water	0.3470 g
**12**	Capsules	Per 2 capsules: green coffee seed extract 350 mg, chlorogenic acids 157.5 mg, maximum caffeine intake 7 mg	2 capsules per day with a little water	0.8115 g
**13**	Tablets	Per 2 tablets: green coffee extract 800 mg, chlorogenic acid 400 mg, caffeine 16 mg	2 tablets 2 times a day before meals	1.9995 g
**14**	Tablets	Per 1 tablet: 116.67 mg green coffee bean extract 60:1	1 tablet twice a day after meals	0.6096 g
**15**	Capsules	Per 1 capsule: decaffeinated green coffee seed extract 400 mg (45% chlorogenic acid), Garcinia Cambogia 80 mg	2 capsules per day before meals	1.2158 g
**16**	Capsules	Per 2 capsules: green tea extract 1370 mg (of which polyphenols 1343 mg, epigallocatechin gallate 604 mg); black pepper extract 20 mg (of which 19 mg piperine)	2 capsules before workout	1.7585 g

Table S3. Statistical and performance characteristics of the proposed method to determine mycotoxins in green coffee-based nutraceutical samples.

Mycotoxin	Linear range (μg/kg)	Linearity (R^2)	LOD (μg/kg)	LOQ (μg/kg)
AFG2	1-100	0.9913	0.3	1
AFG1	1-100	0.9915	0.3	1
AFB2	1-100	0.9934	0.3	1
AFB1	1-100	0.9940	0.3	1

AOH	1-100	0.9932	0.3	1
HT-2	1-100	0.9902	0.3	1
TEN	5-100	0.9948	1.5	5
FB1	1-100	0.9919	0.3	1
T-2	5-100	0.9906	1.5	5
OTA	1-100	0.9924	0.3	1
ZEN	1-100	0.9945	0.3	1
AME	5-100	0.9938	1.5	5
FB2	1-100	0.9862	0.3	1
ENNB1	1-100	0.9834	0.3	1
BEA	1-100	0.9873	0.3	1

Table S4. Recovery and precision assays of the proposed method at two levels of concentration (5 and 50 μg/kg).

	**Recovery (%)**		**Intra-day (% RSD)**		**Inter-day (% RSD)**	
**Mycotoxin**	5 μg/kg	50 μg/kg	5 μg/kg	50 μg/kg	5 μg/kg	50 μg/kg
AFG2	93.4	96.8	9.3	4.3	11.3	5.6
AFG1	91.6	89.2	12.4	8.0	14.0	8.5
AFB2	92.4	90.7	10.9	4.4	10.8	4.9
AFB1	92.4	91.4	8.8	5.1	12.2	6.2
AOH	83.6	76.1	7.8	4.7	13.8	8.6
HT-2	87.9	90.3	9.2	6.1	13.2	7.3
TEN	93.3	104.4	7.0	6.5	12.0	7.2
FB1	87.2	90.1	10.8	8.3	10.2	6.7
T-2	101.1	106.8	7.5	12.5	9.8	10.3
OTA	95.6	105.2	8.2	6.6	11.2	8.3
ZEN	90.6	95.4	5.6	6.8	12.4	8.1
AME	91.8	89.7	6.5	6.3	18.9	12.5
FB2	87.1	89.2	9.1	6.8	19.3	13.5
ENNB1	79.1	80.3	7.1	6.2	9.1	6.8
BEA	115.1	92.5	5.1	4.9	10.1	8.3

toxins MDPI

Table S5. Occurrence study of mycotoxins in green coffee-based food supplements. Results of positive samples (concentration of mycotoxin higher than LOQ. RSD (%). n = 3); <LOQ (detected but not quantified) and <LOD (non detected).

Sample code	AFG2	AFG1	AFB2	AFB1	AOH	H-T2	TEN	FB1	T-2	OTA	ZEN	AME	FB2	ENNB1	BEA
**1**	59.5 (5.6)	<LOD	<LOD	<LOD	<LOD	<LOQ	<LOD	<LOD	<LOD	<LOD	<LOD	<LOD	56.3 (6.1)	<LOQ	4.4 (1.3)
**2**	<LOD	<LOD	<LOD	<LOD	<LOD	<LOD	<LOD	<LOD	<LOD	<LOD	<LOD	<LOD	26.9 (6.5)	<LOD	4.3 (0.1)
**3**	<LOD	<LOD	<LOD	<LOD	<LOD	<LOD	<LOD	<LOD	<LOD	8.7 (1.3)	<LOD	0.2	<LOD	<LOQ	4.5 (2.9)
**4**	<LOD	<LOD	<LOD	<LOD	<LOD	<LOD	<LOD	<LOD	<LOD	<LOD	<LOD	<LOD	4.9 (20.0)	<LOD	4.3 (0.5)
**5**	<LOD	<LOD	<LOD	<LOQ	4.6 (4.0)	<LOD	0.8	<LOD	<LOD	<LOD	<LOD	<LOD	4.7 (6.5)	<LOQ	4.8 (5.7)
**6**	<LOD	<LOD	<LOD	<LOD	<LOD	<LOD	<LOD	<LOD	<LOD	<LOD	<LOD	<LOD	24.5 (1.2)	20.9 (10.0)	19.8 (7.5)
**7**	3.9	<LOD	<LOD	<LOD	<LOD	<LOD	<LOD	21.8	<LOD	<LOD	<LOD	<LOD	14.7	<LOQ	4.4 (0.4)
**8**	<LOD	<LOD	<LOD	<LOD	<LOD	<LOD	0.8 (8.9)	<LOD	<LOD	<LOD	<LOD	<LOD	<LOD	<LOQ	4.5 (3.0)
**9**	<LOD	<LOD	<LOD	<LOD	<LOD	<LOD	<LOD	<LOD	<LOD	<LOD	<LOD	<LOD	15.3 (7.0)	<LOQ	4.3 (0.7)
**10**	25.1 (2.0)	<LOD	<LOD	<LOQ	<LOD	<LOD	<LOD	15.2 (7.2)	<LOD	<LOD	<LOD	<LOD	7.4 (17.8)	<LOQ	3.9 (0.1)
**11**	13.9 (1.4)	<LOD	<LOD	<LOD	<LOD	13.7 (2.2)	<LOD	25.0 (7.4)	<LOD	<LOD	<LOD	<LOD	<LOD	<LOQ	4.0 (1.1)
**12**	<LOD	<LOD	<LOD	<LOD	<LOD	<LOD	<LOD	<LOD	<LOD	9.8 (7.1)	<LOD	<LOD	7.6 (6.8)	<LOQ	4.9 (6.3)
**13**	7.2 (6.1)	<LOD	<LOD	<LOD	<LOD	<LOD	<LOD	<LOD	<LOD	<LOD	<LOD	<LOD	8.1 (12.1)	<LOQ	4.3 (0.1)
**14**	3.7 (18.5)	<LOD	<LOD	<LOD	11.5 (6.4)	<LOD	<LOD	14.4 (2.5)	<LOD	<LOD	<LOD	<LOD	7.3 (3.9)	<LOQ	4.4 (0.6)

**15**	1.5 (28.0)	<LOD	<LOD	<LOD	<LOD	<LOD	<LOD	22.4 (6.4)	<LOD	<LOD	<LOD	<LOD	7.4 (2.0)	<LOQ	4.1 (1.8)
**16**	4.6 (30.9)	<LOD	<LOD	<LOD	12.4	<LOD	0.4 (14)	<LOD	<LOD	<LOD	<LOD	<LOD	<LOD	<LOQ	4.3 (0.0)

Table S6. Consumer groups and mean and 95th percentile (P95) daily herbal based-supplements/nutraceuticals consumption in Italy.

Consumer group	Food consumption	Mean (g/kg bw/day)	P95 (g/kg bw/day)
Adolescents	Green-coffee based supplements	0.10	0.29
Adults		0.11	0.48
Elderly		0.09	0.37

References

1. Chen, W.L.; Chang, C.W.; Chen, C.Y. Measuring Ochratoxin A Concentrations in Coffee Beverages with Immunoaffinity Columns and Ultra-Performance Liquid Chromatography/Tandem Mass Spectrometry. *J AOAC Int* **2016**, *99*, 469–474, doi:10.5740/JAOACINT.15-0233.
2. Armutcu, C.; Uzun, L.; Denizli, A. Determination of Ochratoxin A Traces in Foodstuffs: Comparison of an Automated on-Line Two-Dimensional High-Performance Liquid Chromatography and off-Line Immunoaffinity-High-Performance Liquid Chromatography System. *J Chromatogr A* **2018**, *1569*, 139–148, doi:10.1016/J.CHROMA.2018.07.057.
3. Sueck, F.; Hemp, V.; Specht, J.; Torres, O.; Cramer, B.; Humpf, H.U. Occurrence of the Ochratoxin A Degradation Product 2′R-Ochratoxin A in Coffee and Other Food: An Update. *Toxins 2019, Vol. 11, Page 329* **2019**, *11*, 329, doi:10.3390/TOXINS11060329.
4. Zainudin, B.H.; Iskandar, M.I.; Sharif, S.; Ahmad, A.A.; Safian, M.F. Validation of Quick and Highly Specific Quantitation Method of Mycotoxin in Cocoa Beans by High Resolution Multiple Reaction Monitoring Technique for Reference Materials Analysis. *Journal of Food Composition and Analysis* **2022**, *106*, 104289, doi:10.1016/J.JFCA.2021.104289.
5. Desmarchelier, A.; Tessiot, S.; Bessaire, T.; Racault, L.; Fiorese, E.; Urbani, A.; Chan, W.C.; Cheng, P.; Mottier, P. Combining the Quick, Easy, Cheap, Effective, Rugged and Safe Approach and Clean-up by Immunoaffinity Column for the Analysis of 15 Mycotoxins by Isotope Dilution Liquid Chromatography Tandem Mass Spectrometry. *J Chromatogr A* **2014**, *1337*, 75–84, doi:10.1016/J.CHROMA.2014.02.025.
6. Oueslati, S.; Ben Yakhlef, S.; Vila-Donat, P.; Pallarés, N.; Ferrer, E.; Barba, F.J.; Berrada, H. Multi-Mycotoxin Determination in Coffee Beans Marketed in Tunisia and the Associated Dietary Exposure Assessment. *Food Control* **2022**, *140*, 109127, doi:10.1016/J.FOODCONT.2022.109127.
7. Ouakhssase, A.; Fatini, N.; Ait Addi, E. A Facile Extraction Method Followed by UPLC-MS/MS for the Analysis of Aflatoxins and Ochratoxin A in Raw Coffee Beans. *Food Additives & Contaminants: Part A* **2021**, *38*, 1551–1560, doi:10.1080/19440049.2021.1925165.
8. Mujahid, C.; Savoy, M.C.; Baslé, Q.; Woo, P.M.; Ee, E.C.Y.; Mottier, P.; Bessaire, T. Levels of Alternaria Toxins in Selected Food Commodities Including Green Coffee. *Toxins 2020, Vol. 12, Page 595* **2020**, *12*, 595, doi:10.3390/TOXINS12090595.
9. Nielsen, K.F.; Ngemela, A.F.; Jensen, L.B.; De Medeiros, L.S.; Rasmussen, P.H. UHPLC-MS/MS Determination of Ochratoxin a and Fumonisins in Coffee Using QuEChERS Extraction Combined with Mixed-Mode SPE Purification. *J Agric Food Chem* **2015**, *63*, 1029–1034, doi:10.1021/JF504254Q/ASSET/IMAGES/MEDIUM/JF-2014-04254Q_0003.GIF.
10. Bessaire, T.; Perrin, I.; Tarres, A.; Bebius, A.; Reding, F.; Theurillat, V. Mycotoxins in Green Coffee: Occurrence and Risk Assessment. *Food Control* **2019**, *96*, 59–67, doi:10.1016/J.FOODCONT.2018.08.033.
11. Vaclavik, L.; Vaclavikova, M.; Begley, T.H.; Krynitsky, A.J.; Rader, J.I. Determination of Multiple Mycotoxins in Dietary Supplements Containing Green Coffee Bean Extracts Using Ultrahigh-Performance Liquid Chromatography-

Tandem Mass Spectrometry (UHPLC-MS/MS). *J Agric Food Chem* **2013**, *61*, 4822–4830, doi:10.1021/JF401139U/SUPPL_FILE/JF401139U_SI_001.PDF.
